# Engagement design in studies on pregnancy and infant health using social media: Systematic review

**DOI:** 10.1016/j.pmedr.2020.101113

**Published:** 2020-05-08

**Authors:** Carol Shieh, Israt Khan, Rachel Umoren

**Affiliations:** a Indiana University School of Nursing, 600 Barnhill Drive, Indianapolis, IN 46202, United States; b Indiana University Richard M. Fairbanks School of Public Health, 1050 Wishard Blvd, Indianapolis, IN 46202, United States; c University of Washington, School of Medicine, Department of Pediatrics-Neonatology, 1959 NE Pacific St, Seattle, WA 98195, United States

**Keywords:** Social media, Maternal and infant health, Patient engagement, Systematic review, Pregnancy, Facebook, Interactive engagement, Independent engagement, Health campaign, Social network

## Abstract

Social media utilization is prevalent among reproductive-age women. The literature on how researchers engage women in studies using social media platforms is scarce. This systematic review analyzed participant engagement design in studies using social media and focused on pregnancy and infant health. Methods: A literature search of EBSCO and PubMed databases was conducted. Included studies had to be completed with quantitative data, focus on pregnancy, postpartum or infant health, and use social media in the research process. A matrix of three engagement designs (passive, interactive, independent) and three research processes (recruitment, data analysis, intervention) was used for analysis. Findings: Thirty-one articles that reported 30 studies met the inclusion criteria. Of these, four were randomized controlled trials (RCT), four were non-RCT interventions, and 22 were observational/descriptive studies. The main purpose of using social media was for recruitment (n = 16), data analysis (n = 6), intervention (n = 8), or both recruitment and intervention (n = 1). Passive engagement was a fundamental design approach in all studies to access a data source that was either the participant or the data provided by the participants in social media. Interactive engagement, mostly for recruitment and intervention, was to engage participants in completing study enrollment or in interacting with the study team or fellow participants. Independent engagement involved off-line activities and appeared sporadically in intervention studies. Conclusions: Passive and interactive engagement designs are more frequently used than independent engagement design. Researchers should select suitable designs when studying pregnancy and infant health using social media.

## Highlights

• Passive engagement design is prevalent in pregnancy and infant health studies using social media.
• Interactive engagement design is used for recruitment and intervention for women.
• Independent engagement design is not frequently used to study pregnancy and infant health.

## 1 Introduction

Social media has become the modern day information and communication platform for women of reproductive age. Recent social media utilization data indicate that of the US adults who use social media, 78% are women; over 80% are 18–40 years old; 63% have an annual income of less than $30,000; and 70% are Black or Hispanic (Pew Research Center, 2019a, Pew Research Center, 2019b). Previous studies indicate that women use social media to search pregnancy and parenting information, share information of their choice with others, and build social networks to strengthen social support (Asiodu et al., 2015, Gleeson et al., 2019, Holtz et al., 2015, Pretorius et al., 2019). Because of its potential to reach a large number of women, health experts and institutions have adopted social media as a platform to disseminate health information to the public or a target population (Dyson et al., 2017). Increasingly, researchers also use social media to engage study participants during various stages of research (Gruver et al., 2016).

The concept of patient engagement encourages researchers to make a paradigm shift from studying a health problem without the input of the patient to studying the problem with the patient’s perspective in mind. Patient engagement, often in the form of serving on a study board or advisory council, can increase study enrollment and decrease attrition (Domecq et al., 2014). Patient engagement is gaining importance in healthcare, but there is no accepted definition (Markham et al., 2017). As described in a previous systematic review, patient engagement in eHealth is characterized by three dimensions: behavioral dimension, cognitive dimension, and emotional dimension (Barello et al., 2016). The behavioral dimension consists of engagement activities that enable patients to participate in self-care. The cognitive dimension promotes patient information-seeking and understanding. Lastly, the emotional dimension helps patients connect physiological and emotional reactions, for example, during adjusting to a disease. These three dimensions of patient engagement, however, were used to assess studies on patients with severe medical conditions such as rental transplant, HIV, diabetes, or hypertension.

Esmail and colleagues (2015) proposed three other engagement dimensions (context, process, and impact) for assessing patient and stakeholder engagement. The context dimension is the environment and conditions that support engagement. Availability of training for patients and organization support are examples of this dimension. The process dimension refers to who, when, and how engagement takes place. The impact dimension reflects intended effects, such as long-term or short-term outcomes from the engagement and qualitative or quantitative methods for evaluation. Based on 108 papers, mostly reviews and case studies, Esmail and colleagues (2015), however, found that some papers described impact dimension but none reported context or process dimension.

Lai and colleagues (2019) proposed a matrix for evaluating user engagement in health-specific social media platforms, which comprised five patient engagement categories from the US National eHealth Collaborative Framework (Healthcare Information and Management Systems Society, 2019) and three types of communications tools. The five engagement categories include *inform me*; *engage me*; *empower me*; *partner with me*; and *support my eCommunity*. The three communication tools include those that support one-way, two-way, and multi-way information dissemination and sharing. The matrix contains 15 cells for engagement evaluation. For instance, in one-way communication, users may passively receive health information (inform me), actively select the information they want to know (engage me), or assess risks and benefits of treatment options (empower me). Their matrix has not been used by researchers in systematic review.

Neiger and colleagues (2012) developed a different set of performance indicators for assessing social media in health promotion, including insights, exposure, reach, and engagement. Insights refer to consumer feedback. Exposure is the number of times or impressions that content on social media is viewed. Reach is the number of people who have contact with the social media content. Engagement links social media to actions, which has three levels. Low engagement is merely acknowledging an agreement or preference. Medium engagement involves sharing content with others. High engagement is actual participation in off-line activities.

Extended from Neiger and colleagues’ performance indicators, Platt and colleagues (2016) further developed a framework to evaluate Facebook engagement for public health communication. Their framework has non-user and user engagement factors. The non-user engagement factors include context (social, political, and cultural environment), content (quality and content of advertisements), and setup variables (advertisement type, target audience, and budget). User engagement activities are grouped into six levels. Level 1 is observation measured by the number of people reached by a health campaign. Level 2 is “likes” submitted by the Facebook audience. Level 3 is exploration measured by website clicks on content. Level 4 is connection by sharing content. Level 5 is conversation by posting comments in discussion forums. Level 6 is implementation by engaging in activities outside Facebook. Levels 1, 2 and 3 are considered lower levels of engagement; levels 4 and 5 higher engagement; and level 6 is the highest engagement.

To date, no published systematic review has reported patient engagement design in studies on pregnancy and infant health that incorporate social media. We chose pregnancy and infant health as a focus area for this systematic review for two reasons. As previously mentioned, a large number of social media users are women of reproductive age. Childbearing and childrearing are important health topics for these women (Gleeson et al., 2019, Pretorius et al., 2019). Findings of our systematic review will have potential impacts on designing social media studies focused on this large group of women. Moreover, pregnancy and infant health involves an array of interventions with some targeting general health promotion and others specific disease management. A review of literature on social media research on pregnancy and infant health can enrich our understanding of designing social media studies in response to health promotion and disease management for the pregnant and infant population. The purpose of this systematic review was to analyze participant engagement design in studies that used social media to address pregnancy and infant health issues.

## 2 Methods

### 2.1 Literature search and selection

We define social media as an internet-based social networking platform to facilitate communication, information sharing, and human interaction. We used several key words to search publications in EBSCO and PubMed databases, including (pregnant OR gestational OR maternal OR infant) AND (social media OR Facebook OR Twitter OR Instagram) AND (health campaign OR health promotion OR health). EBSCO consists of MEDLINE, CINAHL Complete, and PsycINFO databases. We further applied filters to narrow the search. These filters included academic journals, English language, full-text, publication years from 2010 to July 2019, and human subjects articles. We limited publication date to 10 years in order to focus on most recent articles. A previous integrative review on eHealth and patient engagement also adopted a 10-year timeframe (Barello et al., 2016). To be included in this systematic review the article had to address pregnancy, postpartum or infant health; report quantitative results from primary or secondary analysis of a study; and include a complete or partial role of social media usage in research. Excluded from our analysis were study protocols, qualitative studies, and studies that focused social media data mining without a mention of pregnancy or infant health, social media as an information-seeking source without participant engagement information, or technology aspect of social media development. We also excluded articles that reported social media in improving communication among care providers.

**Fig. 1:**
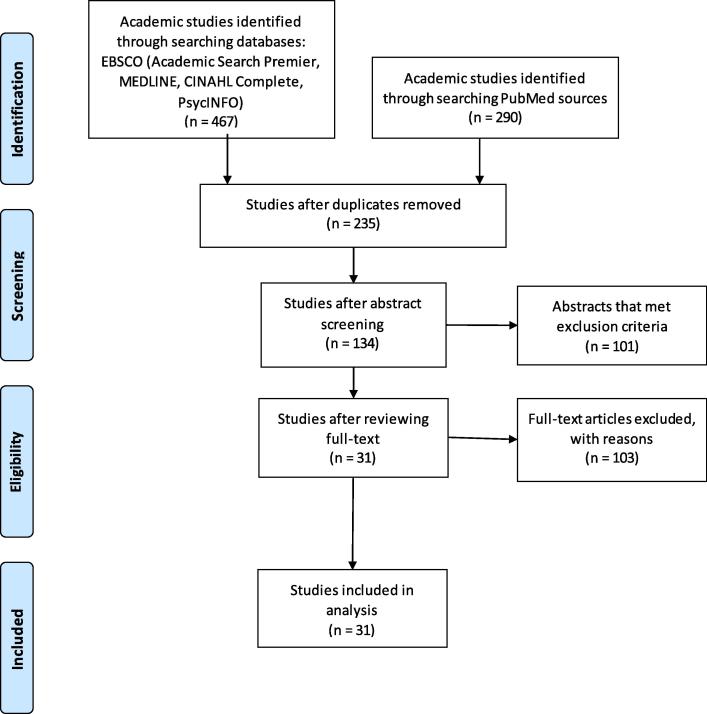
PRISMA Flow Diagram for the Systematic Review of Studies on Pregnancy and Infant Health using Social Media.

As shown in the PRISMA (Fig. 1), 467 articles from EBSCO and 290 articles from PubMed were found. After removal of duplicates, 235 articles remained. Reviewing abstracts against inclusion criteria further eliminated 101 articles. A member of the study team performed the literature search and abstract review. Two study team members read the text of the 134 remaining articles and used the inclusion and exclusion criteria to retain articles. Each article was labeled as “relevant” or “not relevant.” Articles labeled as “not relevant” must have had a reason either of not meeting the inclusion criteria or meeting the exclusion criteria. In this stage, 103 articles were labeled as “not relevant” and were eliminated. The final analysis included 31 articles reporting 30 studies (two articles reported the same study with different study aims). Of the 30 studies, four were randomized controlled trials (RCT), four non RCT interventions, and **22** observational or descriptive studies.

### 2.2 Literature analysis

We developed an assessment matrix that included three engagement designs (passive, interactive, and independent) and three research stages (recruitment, data analysis, and intervention). The three engagement designs were derived from the performance indicators for social media by Neiger et al. (2012) and the Facebook-based engagement framework for public health communication by Platt et al. (2016) but were in a simpler format. We believe our matrix is less cumbersome and suitable for assessing Facebook and non Facebook social media studies relevant to health promotion issues. The matrix by Platt et al. was used for developing a newborn screen and biobank program; therefore, the assessment matrix that we developed for our analysis is likely to be appropriate for evaluating studies on pregnant and parenting women. Definitions of these designs in various research stages and positives/negatives of each design can be found in Table 1.

**Table 1:** Definitions, Positives and Negatives of Participant Engagement Designs in Research Processes.

	Passive Engagement Design	Interactive Engagement Design	Independent engagement Design
Recruitment	To find, inform, and enroll study participants **Examples:** • Paid Ads (Facebook or Google AdWords) • Nonpaid ads	To facilitate study enrollment or completion of study survey **Examples:** • Eligibility screen • Electronic consent • Complete survey	To complete enrollment outside social media **Examples:** • Preforming additional steps to enroll in a study, such as completing enrollment in clinics
Data analysis	To access study participant data in social media **Examples:** • Google search for social media network sites • Twitter hashtag to search for postings • Extraction of Facebook posts	To obtain consent to use participant data **Examples:** • Permission from participants	
Intervention	To retain participants and prevent dropout **Examples:** • Maximize group assignment by IP address • Regularly sending study participants videos, infographics, messages, and photos	To foster interaction of the study participant with the research team or with fellow participants **Examples:** • Blogs, discussion forums, chat rooms, Q/A, virtual one-on-one or group discussion	To engage participants in activities outside the social media platform **Examples:** • Self-monitoring health behavior, going to a focus group meeting or an in-person study orientation, or participant in telephone coach
Positives	• Can use paid or non-paid Ads • Recruitment can be efficient and less costly • Recruitment targeting a specific population • Preset capped budget for recruitment Ads • Used to find lost-to-follow-up study participants • Access existing posts for analysis • Decrease subject dropout	• Can complete recruitment and data collection the same time • Provide support • Can be small group or one-on-one • Can be real time and instant feedback	• Verify study eligibility offline • Increase health behavior • Empower participants to take charge
Negatives	• Falsify eligibility information by social media users • Compete with other ads posted in free social media sites • Permission to access some social media groups may not be granted	• Cyber bullying • Potential abusive language in posting	• Need additional measures to assess off-line activity attendance

We examined each article and identified how engagement designs were utilized for recruitment, data analysis, and intervention. Passive engagement design is used to find, inform, and enroll participants during recruitment, access participant data from social media for data analysis, or retain study participants in intervention. Interactive engagement design facilitates study enrollment during recruitment or fosters interaction of study participants with the research team or with fellow participants during intervention. Independent engagement design encourages participants to engage in activities outside the social media platform, such as preforming additional steps to enroll in a study, self-monitoring health behavior, or going to a focus group meeting during intervention. Two team members extracted information from each article and cross-checked the accuracy of information as shown in Table 3. Inter-rate agreement was 84%. Disagreement was solved by re-examining the articles.

## 3 Results

Our analysis included 31 articles, which contained data from 30 studies (see Table 2). Among the 31 articles, 17 reported studies conducted in the United States, 4 in Canada, 3 in the United Kingdom, 3 in Australia and New Zealand, 1 in Ireland, the Netherlands, and Brazil each, and 1 unknown. Facebook was the most frequently used social media platform (n = 17) followed by Facebook combined with Google, Craigslist, university clinical trial website, local city classifieds or individual study websites (n = 8); Twitter (n = 3); and social media sites created by researchers or special interest groups (n = 3). Health topics reported in the 31 articles varied from gestational weight gain, pregnancy health, abortion, and postpartum anxiety to breastfeeding, infant vaccination, birth defects, contraception, and drinking during pregnancy. The main purpose of using social media was for recruitment (n = 16), data analysis (n = 6), intervention (n = 8), or both recruitment and intervention (n = 1).

**Table 2:** Characteristics of Studies Reported in Articles on Pregnancy and Infant Health (N = 31).

Author Year	Country	Function of Social Media	Primary Social Media	Health Topic	Study Purpose
Adam 2016	Canada	Recruitment for RCT	Facebook	Gestational weight gain, dietary intake	Compared recruitment approaches: Facebook and traditional methods (TV, newsletter, etc.)
Admon 2016	USA	Recruitment for survey	Facebook	Pregnancy health	Compared feasibility and cost of recruitment methods: social media-based and clinic-based approaches
Altshuler 2015	USA	Recruitment for survey	Facebook	Abortion	Employed internet-based recruitment to gather information about young people’s attitudes toward abortion
Arcia 2014	USA	Recruitment for survey	Facebook	Childbirth preference	Evaluated Facebook advertisement to recruit a national sample of nulliparous women in their first 20 weeks of pregnancy
Ashford 2018	UK	Recruitment and intervention	Facebook, Twitter, Web-based intervention site	Postpartum anxiety	Investigated feasibility and acceptability of iWaWa among English postpartum women with anxiety
Byker 2019	USA	Intervention (Social media campaign)	Facebook	Contraception	Explored the effect of an information campaign on social media on long-acting reversible contraception use
Cavalcanti 2019	Brazil	Intervention	Facebook closed group	Exclusive breastfeeding	Evaluated the effectiveness of a participatory intervention using online social network on exclusive breastfeeding during the first six months of the child's life
Daley* 2018	USA	Intervention	Study specific Internet-based social media	Infant vaccination	Assessed the effectiveness of an Internet-based platform with vaccine information and interactive social media components on parents’ vaccine-related attitude.
Emery 2018	UK	Recruitment for intervention	Google and Facebook	Smoking cessation	Explored the uptake and cost-effectiveness of a text message intervention for pregnant smokers (MiQuit) when advertised on the Internet
Fiks 2017	USA	Intervention	Facebook	Infant obesity prevention	Tested a Facebook peer-group intervention Grow2Gether for low-income mothers to foster behaviors promoting healthy infant growth
Glanz* 2017	USA	Intervention	Study specific Internet-based social media	Childhood immunization	Evaluated the effectiveness of web-based social media interventions on early childhood immunization
Golder 2019	Unable to confirm	Data analysis (Identification of women having a baby with a birth defect)	Twitter	Birth defects	Assessed the feasibility of using social media data as an alternative source for pregnancy surveillance for regulatory decision making
Graham 2019	Canada	Recruitment	Google and Facebook	Pregnancy weight gain	Described the implementation of a digital media campaign using Google AdsWords and Facebook to promote healthy pregnancy weight gain
Harpel 2018	USA	Recruitment for survey	Facebook	Pregnancy-related information	Examined the use of Facebook to share pregnancy-related information and prenatal attachment
Harris 2015	Australia	Recruitment for longitudinal study	Facebook, Twitter, forum posts, and study website	Contraceptive use, pregnancy intention, and decisions	Evaluated online recruitment for a longitudinal cohort study
Herbell 2019	USA	Recruitment	Facebook	NR	Evaluated diversified recruitment strategies with the use of Facebook to recruit pregnant women into research
Hether 2016	USA	Data analysis	Two social networking sites (pregnancy forums)	Social support	Identified dimensions of social support exchanged in social networking sites by pregnant women
Holtz 2015	USA	Recruitment for survey	Facebook	Motherhood	Explored motivations for participation in online engagement on motherhood
Laws 2016	Australia	Recruitment for intervention	Facebook	Infant feeding	Compared recruitment rate, costs, and characteristics of participants recruited from various recruitment approaches to an mHealth intervention on infant feeding
MacDonnell 2019	USA	Recruitment	Facebook, Craigslist, Twitter, university clinical trial page	Alcohol use	Identified affordable and efficient methods to recruit women for the Contraception and Alcohol Risk Reduction Internet intervention (CARRII)
Mackert 2012	USA	Intervention	Twitter	Multivitamin intake	Investigated the use of Twitter as a health promotion tool to reach out to young women to promote multivitamins intake
Marshall 2019	USA	Data analysis	Facebook	Weight gain, diet and exercise during pregnancy	Analyzed social media posting related to weight gain, diet, and exercise during pregnancy
Meaney 2016	Ireland	Data analysis	Twitter	Perinatal death	Explored Twitter status updates and subsequent responses relating to a number of perinatal deaths
Moore 2017	UK	Recruitment for survey	Online forum, Facebook, Twitter	Perinatal mental health	Tested a model that measured the mediating role of stigma between online forum use and disclosure of affective symptoms to health care providers
Parackal 2017	New Zealand	Data analysis	Facebook	Drinking during pregnancy	Examined user engagement for a public health campaign via Facebook advertisement that involved a video and three banners
Perrin 2014	USA	Data analysis	Facebook	Human milk sharing	Described the size and activity of online milk sharing and evaluated communication among participants
Shere 2014	Canada	Recruitment	Facebook, Twitter, Craigslist, Kijiji (local online classifieds), and online forums	Folic acid	Compared recruitment success and efficiency between traditional healthcare-based methods of recruitment vs. social media
Silfee 2018	USA	Intervention	Facebook	Weight loss	Evaluated feasibility of a weight loss intervention (Fresh Start) using Facebook for low-income postpartum women
Stephenson 2019	Canada	Recruitment (Re-engaging lost-to-follow-up participants)	Facebook	Parental well-being and outcomes of children	Determined if Facebook was a feasible method for identifying and contacting participants of a longitudinal pregnancy cohort who were lost to follow-up
Van Gelder 2019	Nether-lands	Recruitment for longitudinal study	Facebook and Google AdWords	Prenatal health	Assessed the feasibility of using Facebook advertisements and Google AdWords for recruitment of pregnant women into a prospective cohort PRegnancy and Infant DEvelopment (PRIDE) study with long-term follow-up
William 2019	USA	Enhanced intervention	Facebook	Optimal weight gain	Tested the feasibility of an intervention with mother-infant dyads to promote recommended GWG in primigravidas, optimal infant feeding, and return to pre pregnancy weight

*: Articles are from the same study with different focuses.

As shown in Table 3, of the 17 articles that described using social media to recruit participants, all but two used passive and interactive engagement designs and only one article reported using independent engagement design. For articles with data analysis as a focus (n = 6), passive engagement was the primary design reported in 6 articles and interactive engagement (n = 1) and independent engagement (n = 0) designs were not common. Articles, reporting the use of social media for intervention (n = 9), included passive (n = 9), interactive (n = 8), and independent engagement designs (n = 3) with independent engagement design to a lesser degree.

**Table 3:** Three Types of Engagement Designs used in Various Research Stages.

First Author/Year	Purpose of Social Media	Passive Engagement	Interactive Engagement	Independent Engagement
Adam/2016	Recruitment	x	x	
Admon/2016	Recruitment	x	x	
Altshuler/2015	Recruitment	x	x	
Arcia/2014	Recruitment	x	x	
Emery/2018	Recruitment	x	x	
Graham/2019	Recruitment	x	x	
Harpel/2018	Recruitment	x	x	
Harris/2015	Recruitment	x	x	
Herbell/2019	Recruitment	x	x	
Holtz/2015	Recruitment	x	x	
Laws/2016	Recruitment	x	x	
MacDonnell/2019	Recruitment	x	x	
Moore/2017	Recruitment	x	x	
Shere/2014	Recruitment	x		x
Stephenson/2019	Recruitment		x	
Van Gelder/2019	Recruitment	x	x	
Golder/2019	Data analysis	x		
Hether/2016	Data analysis	x		
Marshall/2019	Data analysis	x	x	
Meaney/2016	Data analysis	x		
Parackal/2017	Data analysis	x		
Perrin/2014	Data analysis	x		
Byker/2019	Intervention	x		x
Cavalcanti/2019	Intervention	x	x	
Daley/2018*	Intervention	x	x	
Fiks/2017	Intervention	x	x	
Glanz/2017*	Intervention	x	x	
Mackert/2012	Intervention	x	x	
Silfee/2018	Intervention	x	x	x
William/2019	Intervention	x	x	
Ashford/2018	Recruitment Intervention	x x	x x	x

* Articles from the same study but reported different components.

### 3.1 Engagement design for recruitment

Passive engagement design for recruitment involved using paid and non-paid social media to reach out to potential study participants (Table 4). Researchers sent advertisements via paid services such as Facebook newsfeed and Google AdWords to potential study participants. Facebook advertisements could target a specific group of women, such as women aged 18–44 residing in the US for a study on childbirth preference among nulliparous women (Arcia, 2014). Google AdWords could also display paid advertisement text to users who happen to use similar search words. For instance, Van Gelder and colleagues (2019) chose 20 search terms related to pregnancy in Google AdWords to recruit pregnant women less than 17 weeks pregnant for a longitudinal study on prenatal health and infant development. When using paid services in passive engagement design, researchers could adopt the lowest cost bid through an automatic bidding/auction system in Facebook and Google AdWords, or a preset capped budget (Arcia, 2014, Graham et al., 2019, Laws et al., 2016).

**Table 4:** Engagement Designs and Costs for Social Media-Based Recruitment Reported in Articles on Pregnancy and Infant Health (N = 17).

Author Year	Passive Engagement	Interactive Engagement	Independent Engagement	Cost
Adam 2016	• Facebook ads received by 44,439 people on their Facebook newsfeed • Ads run on nonconsecutive days to avoid information overload • Traditional approaches: local TV news, fairs, offices	• Women clicked on the bottom link to study website. • A click-through rate (CRT) of 2.3% to the study website. • Facebook Ads: 40/45 screened, 25 eligible, 0.96 eligible participant/day • Traditional approaches: 64/70 screened, 45 eligible, 0.2 eligible participants/day	NR	• Facebook: Canadian $20.28/eligible participant • Traditional: Canadian $24.15/eligible participant
Admon 2016	• Facebook Ads shown on newsfeeds of 364,035 users: image, caption, link to survey • Clinic recruitment: OB visits	• Participants clicked on the hyperlink within the Ad to go to the survey website. • 9972 clicks on the Ads • 1323 entries to the survey • Facebook: Of those who consented, 74.02% eligible and 64.43% completed the survey	NR	• Cost per completed survey: Social media($14.63) Clinic-based ($23.51)
Arcia 2014	• Facebook: 14 Ads, automatic auction to select Ads • Ads viewed 10,577,381 times by 7,248,985 Facebook users • Ads target women aged 18-44 residing in US. • Cost-Per-Click model • Ad selection factors: 1) how many other competing Ads, 2) the maximum per-click bid (e.g., $1.10), and 3) how well the Ad has performed in the past	• After clinking on the Ads, users were led to the study survey welcome page. • The Ads received 6094 clicks by 5963 unique users • A total CTR of 0.06% and a unique CRT of 0.08% • The daily click was 10-70 and averaged 48.4.	NR	• Cost-per-click: $0.15 to $0.94. • Mean cost: $0.63 per click. • Daily cost: $6.84 to $43.63, averaged $30.33. • $11.11 per eligible participant. • $0.63/click
Altshuler 2015	• Facebook Ads targeted at English-speaking , aged 13–29 • Ad materials were intentionally nonspecific (no mention of abortion) to recruit individuals with diverse abortion views. • Study website: For recruited individuals to learn about the study, and determine eligibility	• Participants take the 21-item, multiple-choice Question Pro™-hosted survey on the study website. • Twitter posts and a YouTube video embedded on the study website • Maintained a CRT of 0.05% (8673 total clicks) • 10,600 visitors to the study website with 24–82 visits per day; 1739 survey views; 78% survey completion rate	NR	• The survey ran for 109 days and cost US $3970 for Facebook Ads. • $3 per enrolled participant
Ashford* 2018	• Facebook, Twitter, and UK third-party parenthood websites • Traditional: posters and flyers in two clinical settings in England (hospital and health visiting clinic).	• Study website linked to web-based questionnaire consisting of the electronic informed consent procedure, the eligibility questions and the baseline assessment	NR	NR
Emery 2018	• Google AdWords (search-based), Facebook Ads (banner) • Two non-commercial websites • Separate adverts were created for each of the 4 online settings, with input from a patient and public involvement representative • Text kept as similar as possible between adverts given their character or space limits	• Those wanting to initiate MiQuit had to navigate to the “sign-up” page, click on the “sign-up” button, and submit a response to a question asking where they first heard about MiQuit. • MiQuit was initiated by 93 individuals, 42 from each commercial sites and 9 from the free links. • Uptake (the percentage who subsequently initiated MiQuit after clicking on an advert to the MiQuit website): 3.38%.	NR	• The mean cost per advert-click to the MiQuit website was £1.33 for the Google advert and £0.53 for the Facebook advert.
Graham 2019	• Google AdWords: paid service to display Ad text and a campaign website. • Google AdWords: uses automatic cost per click system to pay only when the Ad was clicked. Budget at Canadian $10/day. • Google AdWords: 43,449 impressions. The average position of a campaign was 1.3 (displayed first or second when an associated keyword was searched). • Facebook Ads: paid messages displayed to predefined members using an automatic bidding system to achieve the highest number of clicks and the lowest cost. Budget at Canadian $26 for Ads run continuously. • Facebook: 772,263 impressions. The average number of times an Ad was displayed to the same individual ranged from 2.53 to 3.28.	• A user clicked on the Ad to go to campaign website which contained healthy weight gain related information, calculator to find BMI and recommended weight gain range. • Google AdWords: clicked 2522 times, CRT of 5.80%. The most popular search term that led to an Ad click was “calculate weight gain during pregnancy” with 137 clicks. The search term “pregnancy weight gain” had the highest CRT at 24.07%. There were 1989 conversions (actions a user completes after clicking on an Ad) representing a 78.9% conversion rate. • Facebook: clicked 14,482 times, CTR of 1.88%. Ads received 43 comments, 28 shares, and 247 reactions. The highest-performing Ad, as determined by the CTR, occurred in the third phase and included the AHS logo, an image of diverse women, and the headline “Pregnancy weight is not the same for every woman.	NR	• Google AdWords: total cost Can $1,913.72, cost per click Can $0.76. • Facebook: total cost Can $5067, cost per click Can $0.35
Harpel 2018	• Facebook advertisements • Researcher’s personal and research Facebook pages • Posts to professional organization listservs	• Individuals interested in the survey were directed through the Ad to the secure web-based survey system. • 5395 clicks on the survey	NR	NR

NR: not relevant *: Social media also used for intervention.

Other researchers used non-paid advertisements, which were free but researchers may request permission to access some social network groups or compete with others who also wanted to post advertisement in the same social media site. For instance, Herbell (2019) obtained permission from 61 Facebook parent discussion private group owners and posted study flyers in those Facebook pages to recruit pregnant women. MacDonnell and colleagues (2019) used free Craigslist advertisement to post study information when recruiting women into their Contraception and Alcohol Risk Reduction Internet Intervention. Shere and colleagues (2014) used drug-sponsored sites, local classifieds, pregnancy discussion forums, and message boards to disseminate study information to recruit women not taking folic acid three months before pregnancy.

Passive engagement could be used to reconnect lost-to-follow-up study participants with a study. Stephenson and colleagues (2019) identified lost-to-follow-up participants from Facebook profiles by names, birth date, home address, email and alternate contact in friends list. These lost-to-follow people were invited back to the study.

Interactive engagement design during recruitment was to engage participants in completing enrollment process. To complete enrollment, potential participants clicked on a hyperlink in a recruitment site to go to a study website. In the study website, women could read detailed study information (Graham et al., 2019), answer questions for eligibility screens (Emery et al., 2018), give electronic consent (Admon et al., 2016, Ashford et al., 2018), send emails to the study team (Harris et al., 2015), or sign up for the study (Van Gelder et al., 2019). In six articles, completing enrollment was demonstrated by participants clicking on a link to fill out surveys or questionnaires (Admon et al., 2016, Altshuler et al., 2015, Harpel, 2018, Harris et al., 2015, Holtz et al., 2015, Moore et al., 2017).

Only one article described using independent engagement design for recruitment, in which study participants met study staff in the hospital to go over consent (Shere et al., 2014).

It was noted that when passive engagement design was used for recruitment, some researchers calculated click through rate (CTR), which is the proportion of women clicked on the advertisement in relation to the women who viewed the advertisement, to estimate their reach to potential study participants. CTR varied among studies ranging from 0.06% to 0.08% (Arcia, 2014) to 5.80% (Graham et al., 2019). Other researchers reported cost when using social media for recruitment. Arcia (2014) reported the **cost per click** when using Facebook advertisement as $0.63. In the studies conducted in the UK and Canada, cost per click for Facebook advertisements (€0.53 and CAD$0.35, respectively) seemed to be cheaper than those for Google AdWorks (€1.33 and CAD$0.76, respectively) (Emery et al., 2018, Graham et al., 2019). Other measures were cost per completed survey (Admon et al., 2016) and cost per eligible participant (Arcia, 2014, Harris et al., 2015, Laws et al., 2016).

### 3.2 Engagement design for data analysis

Six articles described using social media for data analysis (Table 5). All of them reported passive engagement, one addressed interactive engagement, and none described independent engagement. The goal of passive engagement for data analysis was to access data posted in social media by participants. Researchers applied different methods to locate social media data, such as using an automatic classification system to identify pregnant women experiencing a birth defect from Twitter postings (Golder et al., 2019). Meaney and colleagues (2016) used hashtags to capture tweets and re-tweets related to fetal death. Marshall and colleagues (2019) adopted the Facebook application program interface at two time points to capture user postings pertaining to pregnant weight gain, food, and exercise. Perrin and colleagues (2014) classified postings into four groups based on original or reply postings related to breast milk sharing: original offer, original request, reply offer and reply request.

**Table 5:** Engagement Designs for Social Media-Based Data Analysis in Articles on Pregnancy and Infant Health (N = 6).

Author Year	Passive Engagement	Interactive Engagement	Independent Engagement
Golder 2019	• Automatic classification system to identify pregnant women from Twitter postings. • 196 Twitter users identified as birth defect case cohort if postings mentioned a birth defect of their baby. Another 196 as controls if posting without mention of a birth defect. • Timeline of case were matched on timeline of 196 controls	NR	NR
Hether 2016	• Google search to identify 8 social networking sites, 2 general pregnancy forums selected • 704 participants across both sites. These members posted 525 support-seeking messages and 1965 support-providing messages. • Original posts and the first 10 responses for a month period were analyzed.	NR	NR
Marshall 2019	• Extraction of Facebook posts by women recruited from primary clinics was performed using the Facebook application program interface (API) at two separate occasions in 2016—once upon recruitment (usually in the 1st and 2nd trimesters) and again in the 3rd trimester. • Posts pertaining to weight gain, food and exercise were identified by finding keywords (e.g., craving, food, fat, exercise) and their morphological variants using Natural Language Processing (NLP).	• Participants granted data collectors access to their accounts by signing into Facebook during a standard clinic visit. Access was immediately terminated after the participants logged out of their accounts. • Of all mined posts (n = 2899), 311 included information relating to health behaviors in pregnancy.	NR
Meaney2016	• Twitter: Hashtag and terms related to “fatal failures“ or fetal death were used to search posting. • Tweets, re-tweets, and replies • Of the 3577 Twitter status updates, 45.15% were tweets, 38.92% were retweets, and 15.94% were replies.	NR	NR
Parackal 2017	• Facebook advertising to deliver public health messages: a video and three banner advertisements • Meta data from Facebook for analysis • Facebook comments: extraction required expanding the conversation threads to make all the comments visible. • The video had 203,754 views and the promotional materials (video and banner Ads) generated 819 comments, 6125 likes, and 300 shares. • This campaign evoked all three sentiment valences: positive, neutral, and negative. Proportions of negative comments were higher than the positive and neutral comments.	NR	NR
Perrin 2014	• Facebook search engine to do initial search Human Milk for Human Babies (HM4HB) and Eats on Feets (EOF) groups in the United States. • Use the number of Facebook ‘‘likes’’ as a measure of community size. The median number of ‘‘likes’’ per state was 680 (interquartile range, 379–1151) for HM4HB groups • 9 HM4HB communities were selected for the analysis: 3 in the first quartile (small), 3 in the second or third quartile (medium), and 3 in the fourth quartile (large). • 954 individuals participating in the milk sharing communities • 4 classifications of posts: original offer (an original post by an individual offering her milk), an original request (an original post by an individual asking for milk), a reply offer (a reply to an original request indicating an interest in giving milk), or a reply request (a reply to an original offer indicating an interest in receiving milk.	NR	NR

NR: Not relevant.

The one article that addressed interactive engagement design for data analysis involved asking study participants to grant data collectors access to their Facebook accounts (Marshall et al., 2019).

### 3.3 Engagement design for intervention

Nine articles including eight studies reported using social media for intervention (Table 6). Almost all of them used passive and interactive engagement designs but only three demonstrated independent engagement design. Passive engagement design for intervention mostly was to maximize the accuracy of reach to potential participants and to retain enrolled participants. Some researchers used zip codes to select women into the treatment group and to verify the accuracy of selection with the IP address and friends profile locations in Facebook (Byker et al., 2019). Other researchers assigned women into small Facebook groups to facilitate retention (Fiks et al., 2017). Passive engagement design to retain participants also included regularly posted videos, infographics, messages, and photos on websites or Facebook to update study information for participants (Cavalcanti et al., 2019, Silfee et al., 2018).

**Table 6:** Engagement Designs for Social Media-Based Interventions in Articles on Pregnancy and Infant Health (N = 9).

Author Year	Passive Engagement	Interactive Engagement	Independent Engagement
Ashford* 2018	Module view: Web-based self-help treatments, including 1 module of concept explanations	• Interactive component: with multimedia presentations and interactive material • A link to each module on a password-protected blog page of the iWaWa study website. • Study website: web-based questionnaire	• 7 web-based modules of practice • Optional 30-minute telephone coach support with weekly practice module
Byker 2019	• Contraceptive information campaign carried out on social media. • Facebook users in the treatment areas (zip codes) received 3 informational Ads on efficacy, easy-of-use, and safety. • Facebook: residence based on the “current city” validated with IP address and friends' profile locations. • About 80% of the targeted population saw at least one advertisement. • 82% of the Ads appeared in the desktop right column, 12% appeared in the mobile newsfeed, and 6% appeared on third-party mobile apps and websites. • Facebook accurately targeting these advertisements by zip codes: 87.4% of respondents from the study region self-reported zip codes in the intended treatment group.	NR	• Women went to one of the 21 Planned Parenthood of Northern New England Health Centers to receive long-acting reversal contraception.
Cavalcanti 2019	• Mothers were recruited from maternal ward of a hospital • Facebook closed group called Projeto Amamenta Mamãe (Mama Breastfeeding) for 24 weeks. • The women were tagged in a post of the group based on a weekly topic	• Each mother received a virtual invitation that she should accept to allow her inclusion in the intervention group (IG). • The post had messages of encouragement and clarification. • A conversation to raise doubts, comment on something, or share their experiences on the specific topic of the poster. • Each tag or participation in the group generated an automatic and immediate notification on the personal profile of each woman, enabling communication in real time.	NR
Daley** 2019	• Electronic health records to identify potential participants • Monthly newsletter via email: encouraging website use, highlighting website updates, and providing additional vaccine content. • Vaccine Information (VI) group accessed the website but no interaction • Usual care group	• A blog, discussion and chat room: Vaccine information and social media interactive (VSM) group access these via the study website. • 1 or 2 blog posts per month: covering timely or controversial issues such as new vaccine safety research, recent vaccine-preventable disease outbreaks, or changes in state or national immunization policies. • An “Ask a Question” portal: VSM participants could direct questions to vaccine experts (a vaccine safety researcher, a pediatric infectious diseases specialist, a general pediatrician, and a risk communication specialist). • For any questions submitted privately through the portal or by e-mail, personalized responses were provided within 2 business days • Real time monthly online chat sessions: VSM group could converse with a team of vaccine experts. • All interactive website and social media components were moderated to prevent bullying, abusive language, and disclosure of personal health information. • Surveys administered online using a secure platform. • Up to 8 email reminders if survey not done.	NR
Fiks 2017	• Women recruited from two high-volume OB clinics • Medical records to prescreen women • Text messaging reminder to all but the control group did not join Facebook interaction • Weekly video based curriculum (infant feeding, sleep, positive parenting, maternal wellbeing, etc.) and written posts	• Facebook private group of 9–13 women from 2 months before delivery to infant age 9 months • 4 separate peer groups involved in video based curriculum and participant interaction • Discussing parenting topics, sharing photos and questions, providing feedback to each other and receiving feedback from facilitator. • Rule: must post once in the group to receive monthly stipend	NR
Glanz** 2017	• Electric medical records to identify subjects • Postcards, emails and phone calls to elicit participation • Vaccine information on website	• Blog, discussion forum, chat room and “Ask a question” portal. • Participants could directly ask our experts questions about vaccination and contribute comments.	NR
Mackert 2012	• Recruit female undergraduate students from a southwestern university in exchange for extra credit. • Each message was displayed as a tweet from a Twitter account.	• Experimental group exposed to 9 multivitamins promotion messages presented in a randomized order. • Participants re-tweeted messages. • The study employed a self-administered online survey as the data collection method.	NR
Silfee 2018	• Recruitment from WIC program and electronic records • Videos and pictures from the in-person protocol were included in posts where applicable and supplemented by additional photos, infographics, and videos extracted from web-based sources with special attention to maintaining the original message.	• First 8 weeks: Facebook intervention posts were delivered 2 times per day, with additional posts from coaches aiming to stimulate interaction among participants or respond to participants' questions and challenges. • For the following 8 weeks: posts were delivered once per day without additional coaching. • To promote interaction among participants in the Facebook group, all posts ended with an open-ended question regarding the topic of the post • Tasks of intervention coach and assistant coach included liking and commenting on the women's posts or comments, encouraging discussion and sharing of strategies to deal with challenges to goal attainment or weight loss among the women, answering questions, and providing support.	• The intervention consisted of an 8-week intervention phase followed by an 8-week maintenance phase delivered via a secret, private Facebook group, preceded by a 90-minute in-person orientation session
William 2019	• An automatic text message system: provided updates and feedback parentally • Recruitment via Federally Qualified Health Center (FQHC) located near the U.S.-Mexico border in San Diego • Participants recruited during first prenatal visit • Post messages in a Facebook page	• A private group Facebook® page to post messages and provided interaction between the *promotora* (community health worker) and participants postnally. • In order to join the private Facebook® group, participants maintained an active Facebook® user account and became Facebook® “friends” with the *promotora*.	NR

NR: Not relevant *: Social medial also used for recruitment **: Articles from the same study but reported different components.

Interactive engagement design for intervention had two functions: participant interaction with the study team and interaction among fellow participants. Interaction could be done via blogs, discussion forums or chat rooms. In some studies, a facilitator would invite participants to a virtual discussion or support group, lead group discussion, and communicate one-on-one with each individual participant. In the study conducted by William and colleagues (2019), the promotora (community health worker) invited Spanish-speaking participants to a Facebook group and provided one-on-one interaction to promote optimal gestational weight gain and infant feeding. In Cavalcanti and colleagues’ study (2019), women in the intervention group were invited to join a closed Facebook group called Projeto Amamenta Mamãe (Mama Breastfeeding) where women could raise doubts, make comments, and share experiences with each other. Interactive engagement design also included real time and instant feedback. For instance, women in a Facebook study group on exclusive breastfeeding received an automatic and immediate notification on the personal profile of each woman, enabling communication in real time (Cavalcanti et al., 2019).

Independent engagement for intervention aimed to engage participants in off-line activities. Ashford and colleagues (2018) asked study participants to do seven weekly practice modules to promote mental health and have 30 min of telephone coach support weekly outside social media engagement. Byker and colleagues (2019) delivered Facebook health campaign about long-term reversible contraception to women in treatment locations and then measured independent engagement in getting this contraception in clinics. Silfee and colleagues (2018) asked women before joining a Facebook group to engage in a 90-minute in-person orientation session.

## 4 Discussion

In this systematic review, we analyzed passive, interactive, and independent engagement designs for recruitment, data analysis, and intervention in 31 articles that reported pregnancy and infant health studies involving social media. Major findings from our review included (1) passive engagement design was prevalent in all studies and was utilized at various research stages, including recruitment, data analysis, and interaction; (2) interactive engagement design was common for recruitment and intervention; and (3) independent engagement design was used the least and it appeared mostly during intervention.

Although passive engagement is a term relative to the participant’s perspective, it is a fundamental design initiated by researchers to access a data source, whether that is the participant or the data posted by the participant in social media, as well as to retain participants in a study. We found that researchers used passive engagement design through paid or non-paid social media platforms to reach out to potential participants. For data analysis, passive engagement design enabled researchers to access the participant’s existing data already posted in social media. For intervention, passive engagement involved regularly sending participants study related information to increase retention.

A unique passive engagement design in social media research identified was the use of commercial functions in social media to recruit study participants. Paid advertisements in Facebook and Google AdWords could provide pre-selected parameters such as subject demographics, geographic locations, search words, and budget to facilitate recruitment (Emery et al., 2018, Graham et al., 2019, van Gelder et al., 2019). We also found that many researchers believed that social media platforms were a quick way to recruit participants for survey studies (Admon et al., 2016, Arcia, 2014). Shere and colleagues (2014), using interruptive time-series analysis, even identified social media as a significant reason for the increase in recruitment in their study. A disadvantage of using social media for recruitment is the potential for fraudulent application submissions by social media users (MacDonnell et al., 2019). However, researchers may avoid the problem by adopting interactive or independent engagement design. Potential participants can answer eligibility questions online or meet with study staff to complete the eligibility assessment.

Using social media to reengage lost-to-follow-up study participants is an innovative passive engagement design. Stephenson and colleagues (2019) used passive engagement design in their longitudinal study to find study participants who could not be contacted after numerous attempts by phone or email. One reason to support this approach was that participants often used Facebook URLs longer than email addresses or phone numbers. Subject attrition is a common problem especially for longitudinal studies. Future research may further explore the impact of social media on retention, such as how different social media platforms work to reengage lost subjects.

Interactive and independent engagement designs require active participation of the participant. Our analysis found various interactive engagement designs especially for participants to complete study enrollment, fill out surveys, or participate in intervention. For instance, participants could click on a Facebook advertisement to link to a study website in order to fill out a survey (Altshuler et al., 2015) or to send a message to trigger automatic support (Emery et al., 2018). Interactive engagement also occurred between the research team and the participant or among fellow participants particularly in intervention studies. Cyber bullying may potentially be a problem during online interaction. Many researchers set up rules to prevent bullying and abusive language in posting (Daley et al., 2018). This practice should be encouraged for all studies using Facebook platform or other study-specific websites. The intention of engaging participants in online dialogue is to share personal thoughts and opinions. Negative postings, if not regulated, may result in the opposite effect.

Based on our review, independent engagement design was not frequently adopted by researchers. Only four articles described this design and three of them involved intervention**.** It is likely that intervention studies do need participants to engage in off-line activities in order to induce actual behavior change; therefore independent engagement design was mostly adopted in interventions. Additional measures also have to be developed in order to assess actual off-line activity attendance, which could have limited its utilization by researchers. Future research may compare health behavior outcomes of study participants that engage in off-line activities versus those without such activities. Furthermore, future research may investigate lurkers’ (i.e., who read postings but do not post) reactions to off-line activities compared to those of active participants. Many studies in our review reported click through rates or cost per click. This represents another area for future research, as a range for these values, relevant to study design or sample size, has not been established.

### 4.1 Strengths and limitations

To our knowledge, our review is the first systematic analysis to report engagement design in social media-based studies on pregnancy and infant health. The evaluation framework used in our analysis, including passive, interactive, and independent engagement designs, is simple and easy to understand. Our framework was derived from previous studies for Facebook engagement and other social media (Neiger et al., 2012, Platt et al., 2016) but in a much simpler format for assessing Facebook and non Facebook social media. Another unique strength of our review is identifying the application of these engagement designs in the research process, which is different from previous reviews. Depending on project purpose, a researcher may adopt a suitable engagement design to enhance recruitment, analysis, or intervention. A limitation in our review, however, is that we included studies of various pregnant and infant health topics and publication years of only 2010–2019. Our findings are not generalizable to a specific health topic and are limited to findings from the past decade only. Another limitation is that we did not analyze effects associated with each engagement design. For instance, we did not compare recruitment results when using passive engagement designs with paid or non-paid social media platforms. Although we report various engagement designs in our review, we are unable to conclude which is more effective than the others. Furthermore, studies included in our review adopted Facebook and other Internet-based platforms. We did not separate engagement designs in Facebook from those in non-Facebook platforms. Social media use is influenced by availability of technology, geographical locations, and race/ethnicity. Over one half of the articles in our review reported studies conducted in the US. Our review findings therefore may not have a global implication.

## 5 Conclusions

Social media-based studies on pregnancy and infant health utilize various engagement designs during different stages of research. Regardless of study purpose, passive engagement design is commonly used for recruitment and retention of study participants, and for access to data in social media. Interactive and independent engagement designs encourage active participation of the participant. These designs are often used for interventions. Choosing a suitable design may enhance the probability of success in recruitment, data analysis, and intervention.

## Declaration of Competing Interest

The authors declare that they have no known competing financial interests or personal relationships that could have appeared to influence the work reported in this paper.
